# Delivery Mode, Duration of Labor, and Cord Blood Adiponectin, Leptin, and C-Reactive Protein: Results of the Population-Based Ulm Birth Cohort Studies

**DOI:** 10.1371/journal.pone.0149918

**Published:** 2016-02-22

**Authors:** Chad A. Logan, Larissa Thiel, Rebecca Bornemann, Wolfgang Koenig, Frank Reister, Hermann Brenner, Dietrich Rothenbacher, Jon Genuneit

**Affiliations:** 1 Institute of Epidemiology and Medical Biometry, Ulm University, Ulm, Germany; 2 Department of Internal Medicine II—Cardiology, University Medical Center Ulm, Ulm, Germany; 3 Department of Gynecology and Obstetrics, University Medical Center Ulm, Ulm, Germany; 4 Division of Clinical Epidemiology and Aging Research, German Cancer Research Center (DKFZ), Heidelberg, Germany; 5 ‘In-FLAME’ the International Inflammation Network, World Universities Network (WUN), Ulm, Germany; Virgen Macarena University Hospital, School of Medicine, University of Seville, SPAIN

## Abstract

**Background:**

Numerous studies have reported associations between delivery mode and health outcomes in infancy and later life. Previous smaller studies indicated a relationship between delivery mode and newborn inflammation potentially constituting a mediating factor. We aimed to determine the influence of delivery mode and duration of labor on cord blood concentrations of adiponectin, leptin, and high-sensitivity C-reactive protein (hs-CRP).

**Methods:**

In the Ulm SPATZ Health Study, 934 singleton newborns and their mothers were recruited during their hospital stay in the University Medical Center Ulm, Southern Germany, from 04/2012-05/2013. Inflammatory biomarkers were measured by ELISAs (n = 836). Delivery mode was analyzed categorically (elective cesarean (reference), active labor delivery: emergency cesarean, assisted vaginal, and spontaneous vaginal); duration of labor continuously. Following log-transformation, linear regression was used to estimate geometric means ratios (GMR) adjusted for potential confounders for the effects of delivery mode and duration of labor on each biomarker separately. Independent replication was sought in the similarly conducted Ulm Birth Cohort Study recruited from 11/2000-11/2001.

**Results:**

Individually, active labor delivery modes as well as increasing duration of labor were associated with higher leptin and hs-CRP concentrations. After mutual adjustment, the associations with delivery modes were attenuated but those for duration of labor remained statistically significant (GMR (95%CI) 1.10 (1.00; 1.21) and 1.15 (1.04; 1.27) for leptin and hs-CRP per hour of labor, respectively). No significant adjusted associations were observed between delivery modes and adiponectin concentrations. These findings were replicated in an independent birth cohort study.

**Conclusions:**

Cord blood leptin and hs-CRP concentrations were associated with duration of labor rather than delivery mode. Further research is warranted to investigate these associations with additional cytokines involved in inflammatory response to delineate the inflammatory profile. Subsequently, research on determinants of these associations and their role in development of chronic disease is needed.

## Introduction

Numerous studies have identified possible associations between delivery mode and a variety of health outcomes in childhood and later life including overweight and obesity,[1,2] type 1 diabetes[3], asthma,[4–6] atopic dermatitis,[7] allergic sensitivity,[8] celiac disease,[9] and inflammatory bowel disease[10]. One potential biological mechanism which may link the singular event of childbirth to some of these diseases could involve differences in the gut microbiota of cesarean born infants.[11–14] Nevertheless, delivery mode may also serve as a proxy for other risk factors present during fetal development or around the time of birth which may contribute to the association.[15–17]

Several studies have identified associations between adiponectin, leptin, or CRP levels as biomarkers of inflammation in cord blood and early indicators of chronic disease including incidence of lower respiratory tract infections and wheeze,[18,19] infant growth rate,[20] as well as, weight gain and adiposity.[21] These studies suggest that inflammatory processes triggered by delivery itself or by events occurring during the prenatal period may be another possible connection between delivery mode and subsequent disease. However, only three previous studies have principally investigated associations between elective cesarean delivery compared to active labor delivery modes (spontaneous and assisted vaginal and emergency cesarean) and these biomarkers in either cord blood or maternal serum.[22–24] Due to limited sample size and design limitations, these studies were not able to account for multiple potential confounders. Furthermore, it remains unclear whether differences are limited to onset of labor or if they also exist between active labor delivery modes.

To close these gaps, we aimed to determine whether associations exist between delivery modes and cord blood adiponectin, leptin, and CRP concentrations while adjusting for several confounders within the context of a large population-based birth cohort study. A further objective was to evaluate the roles of duration of labor, anesthesia, and induction of labor as potential mediators of these associations. Independent replication was sought in a second birth cohort study.

## Methods

The Ulm SPATZ Health Study was approved by the ethics board of Ulm University (No. 311/11). The Ulm Birth Cohort Study (UBCS) was approved by the ethics boards of Ulm University (No. 98/2000) and of the Physicians’ Boards of the states Baden-Wuerttemberg and Bavaria. Participation was voluntary and written informed consent obtained in each case.

### Study design and population

In the Ulm SPATZ Health Study newborns and their mothers were recruited during their hospital stay following delivery in the University Medical Center Ulm, Southern Germany, between 04/2012 and 05/2013.[25] Exclusion criteria were outpatient delivery, maternal age <18 years, insufficient knowledge of the German language, and transfer of the newborn to intensive care immediately after delivery. At baseline, the cohort included 1,006 newborns of 970 mothers (49% of eligible families, 934 singletons and 36 twin pairs).

In the Ulm Birth Cohort Study newborns and their mothers were recruited during their hospital stay following delivery in the University Medical Center Ulm between 11/2000 and 11/2001.[26] Exclusion criteria were outpatient delivery, maternal age <18 years, insufficient knowledge of the German, Russian or Turkish language, gestational age <32 weeks, birthweight <2500g and transfer of the newborn to intensive care immediately after delivery. At baseline, the cohort included 1,090 newborns of 1,066 mothers (67% of eligible families, 1,042 singletons and 24 twin pairs).

For the purposes of this analysis, the study populations were restricted to singletons. Exposure, outcome, and confounder definitions as well as statistical methods were identical for both studies unless specifically stated otherwise.

### Exposure definition

Delivery mode, duration and medical induction of labor, and administration of anesthesia were ascertained from electronic hospital records. For analysis purposes, delivery mode was consolidated into the following categories (i) elective cesarean section, (ii) vaginal spontaneous, (iii) emergency cesarean [secondary cesarean, emergency cesarean section, or express cesarean section], or (iv) assisted vaginal delivery [vacuum extraction or forceps]. All delivery modes except elective cesarean section followed onset of labor and were thus active labor deliveries. Duration of labor was estimated as a continuous variable based on the time of onset of labor which was self-reported by the mother at the time of admission to the hospital and time of birth recorded by the hospital. Duration was imputed as zero if the mother gave birth by elective cesarean section. In SPATZ, one child was excluded due to missing delivery mode information. Labor was considered induced if hospital records reported the mother receiving prostaglandin (or misoprostol), castor oil, or oxytocin. Administration of anesthesia was assumed if any of the following procedures were reported: single shot or catheter spinal anesthesia, pudendal nerve block, epidural catheter, general anesthesia, or patient controlled analgesia (PCA) pump with remifentanil.

### Outcome definition

Cord blood was collected in S-Monovette 7.5 ml serum-gel tubes (Sarstedt AG & Co, Nümbrecht, Germany), by midwives or obstetricians shortly after delivery, centrifuged, and stored in a refrigerator until further processing and long-term storage at -80°C by trained study personnel. In SPATZ, average time until long-term storage was 2.4 days (sd = 0.8 days). Adiponectin, leptin (both R&D Systems GmbH, Wiesbaden, Germany), and high-sensitivity CRP (hs-CRP, Immunodiagnostik AG, Bensheim, Germany) concentrations were measured by ELISA (SPATZ: n = 837; UBCS: n = 900). Biomarker measurements reported as below detection limit were imputed to the lower detection limit. Hs-CRP levels above 200 μg/L have been associated with amniotic fluid infection, therefore subjects with hs-CRP measurements above 200 μg/L (SPATZ: n = 23; UBCS: n = 53) were excluded from the analysis.[27]

**Fig 1:**
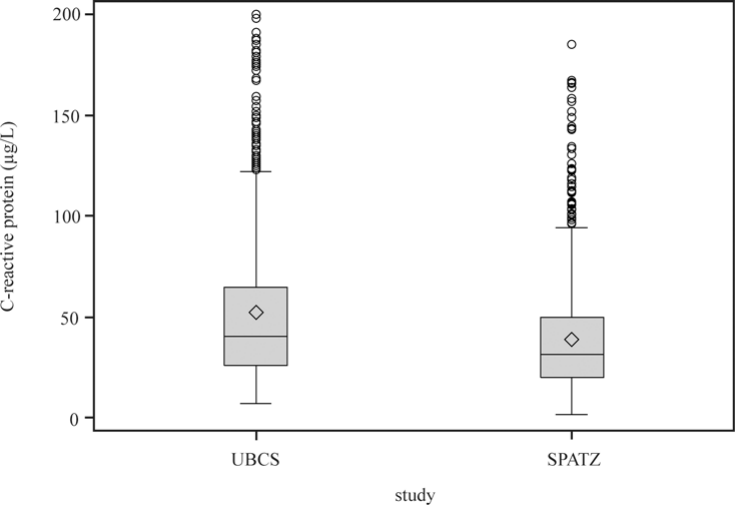
High-sensitivity C-reactive protein distribution in UBCS and SPATZ. ^◊^ arithmetic mean value.

For UBCS, adiponectin and leptin were measured in 06-07/2005 in Heidelberg, Germany, whereas hs-CRP was measured in 08-10/2009 in a research lab at Medical Center Ulm. For SPATZ, all three markers were measured in the latter research lab in 07-08/2013 with the same equipment and by the same technician as in UBCS. For SPATZ, median duration of long-term storage of frozen cord blood was 300 days (25^th^ percentile: 211, 75^th^ percentile: 392) and was correlated with adiponectin concentration (r_Spearman_ = -0.18, p<0.001). Therefore we standardized values by regressing adiponectin concentration against duration of storage using local polynomial regression (PROC LOESS, SAS 9.3). Hs-CRP levels differed between both studies with the whole distribution shifted by approximately 10μg/L towards higher levels in UBCS (Fig 1). With identical lab and technician as well as similar standard curves and controls in both studies we attribute this to the longer duration of long-term storage in UBCS (up to 9 years vs. up to 1.25 years in SPATZ) with evaporation and subsequent concentration of serum.

### Putative confounding variables

Demographic data were collected using a self-administered maternal questionnaire during the hospital stay following delivery. Clinical data related to the child’s delivery were obtained from electronic hospital records. Clinical data related to the mother’s pregnancy were additionally obtained from routine paper documentation; obstetricians in Germany are required to issue to their patients when pregnancy is clinically established and which are generally updated at each clinical visit during pregnancy.

Variables considered as potential confounders pertaining to the child included gender [male, female], birthweight [continuously or categorized as <3000g, 3000g-3499g, 3500g-3999g, ≥4000g], gestational age [weeks], birth season [winter (Dec-Feb), spring (Mar-May), summer (Jun-Aug), fall (Sept-Nov)], cord blood-pH [continuously], base excess [continuously], and APGAR score at 1 minute after delivery [≥7; <7]. Potential confounding variables considered pertaining to the mother included duration of school education [>11 years, ≤11 years], smoking during pregnancy [yes, no], frequency of maternal alcohol consumption in the year before pregnancy [units per week 0, <1, 1, or >1; 1 unit = 0.5L beer, 0.25L wine, 0.1L sparkling wine, or 0.2L schnapps], first trimester body mass index (BMI) [kg/m^2^], preeclampsia [yes, no], maternal age [continuously], and parity [number of previous births of a fetus ≥24 weeks gestation categorized as 0, 1, or >1]. For SPATZ only, HbA1C measured during the hospital stay after delivery [categorized as <5.7 or ≥5.7] was additionally available. First trimester BMI was calculated based on weight measured during the mother’s first obstetric appointment at which pregnancy was clinically established if the appointment took place prior to week 15 of pregnancy (SPATZ: n = 805, mean = 9 wks, sd = 2 wks; UBCS: n = 854, mean = 8.4wks, sd = 2.6wks) or self-reported weight before pregnancy (SPATZ: n = 19; UBCS: n = 46).

### Statistical analyses

In order to assess whether the study populations were representative for the full study cohort, 95% confidence intervals were calculated for proportions within categorical variables and means of continuous variables for all demographic characteristics of the full cohort. Demographic proportions and means of the corresponding variables within the study population were then compared to determine if any characteristics contained values outside of the confidence limits.

Independent associations of putative confounders with each inflammatory marker were tested using Pearson chi-square tests, Kruskal-Wallis tests, and Spearman correlation tests to assess categorical-categorical, categorical–continuous, and continuous-continuous variable pairs, respectively. Putative confounders changing the effect component of the crude geometric means ratio of more than one main exposure associated with at least one inflammatory marker by more than 15% were accounted for as covariates in all models independent of the respective inflammatory marker. Child gender was included in all models although it did not fulfill the above criteria.

Following log-transformation due to the right skewed distribution of the inflammatory markers, linear regression was used to estimate adjusted geometric means ratios (GMR) for the associations of delivery mode and duration of labor (per additional hour) separately with each biomarker. Models were then mutually adjusted for delivery mode and duration of labor to investigate the contribution of both variables to the observed associations. Further analyses restricted to vaginal spontaneous deliveries were conducted to analyze the effects of medically induced labor and anesthesia. Observations with missing data were excluded from adjusted analyses. All statistical analyses were performed with SAS® 9.3 (The SAS Institute, Cary, NC, USA).

## Results

Tables 1 (SPATZ) and 2 (UBCS) display demographic details of the full study cohort compared to the study population included in the analysis dataset. The SPATZ study population contained lower proportions of children delivered by emergency cesarean and with birthweight below 3000g. Consequently, mean birthweight, body length, and gestational age were higher and the proportion of mothers who received anesthesia was slightly lower in the study population. These differences were primarily attributable to exclusion of twin births. The proportions of missing data for any given variable did not significantly differ between full cohort and study population (data not shown).

**Table 1:** Characteristics of the SPATZ study population.

	Total Population (N = 1006)			Study Population (n = 813) ^1^		
Factor	N	% or mean	(95%CI)	n	% or mean	(95%CI)
**Cord blood inflammatory markers**						
Adiponectin (mg/L)	891	30.6	(29.7; 31.4)	813	30.8	(30.0; 31.7)
Leptin (μg/L)	891	10.3	(8.6; 11.9)	813	9.7	(9.1; 10.3)
hs-CRP (μg/L)	866	39.0	(37.1; 40.9)	813	38.5	(36.6; 40.4)
**Delivery mode**						
Vaginal spontaneous	639	63.5%	(60.5%; 66.5%)	547	**67.3%**	(64.1%; 70.5%)
Elective cesarean	125	12.4%	(10.4%; 14.5%)	95	11.7%	(9.5%; 13.9%)
Emergency cesarean	156	15.5%	(13.3%; 17.7%)	96	**11.8%**	(9.6%; 14.0%)
Vaginal assisted	85	8.4%	(6.7%; 10.2%)	75	9.2%	(7.2%; 11.2%)
**Duration of labor (hours)**	952	7.6	(7.2; 8.0)	780	7.8	(7.4; 8.3)
**Anesthesia (during delivery)** ^**2**^	241	37.7%	(34.0%; 41.5%)	197	36.0%	(32.0%; 40.0%)
**Induced labor delivery** ^**2**^	164	25.7%	(22.3%; 29.1%)	134	24.5%	(20.9%; 28.1%)
**Gender**						
Male	523	52.0%	(48.9%; 55.1%)	432	53.1%	(49.7%; 56.6%)
Female	483	48.0%	(44.9%; 51.1%)	381	46.9%	(43.4%; 50.3%)
**Gestational Age (weeks)**	1005	38.8	(38.6; 38.9)	813	**39.0**	(38.9; 39.1)
**Birthweight (g)**	1005	3278.2	(3245.2; 3311.3)	813	**3361.6**	(3328.7; 3394.5)
**Cord blood pH**	989	7.3	(7.3; 7.3)	802	7.3	(7.3; 7.3)
**Base excess**	981	-3.3	(-3.5; -3.2)	796	-3.4	(-3.6; -3.2)
**Maternal age (years)**	1006	32.7	(32.4; 33.0)	813	32.7	(32.3; 33.0)
**Maternal nationality**						
Germany	852	84.7%	(82.5%; 86.9%)	691	85.0%	(82.5%; 87.4%)
Other	144	14.3%	(12.1%; 16.5%)	113	13.9%	(11.5%; 16.3%)
**Parity**						
0	547	54.4%	(51.3%; 57.5%)	426	52.4%	(49.0%; 55.8%)
1	342	34.0%	(31.1%; 36.9%)	291	35.8%	(32.5%; 39.1%)
> 1	116	11.5%	(9.6%; 13.5%)	96	11.8%	(9.6%; 14.0%)
**Maternal BMI (first trimester)**	975	24.8	(24.5; 25.2)	789	25.0	(24.6; 25.4)
**Maternal education**						
> 11 years education	577	57.4%	(54.3%; 60.4%)	477	58.7%	(55.3%; 62.1%)
≤ 11 years education	410	40.8%	(37.7%; 43.8%)	321	39.5%	(36.1%; 42.8%)
**Smoking during pregnancy**	72	7.2%	(5.6%; 8.7%)	62	7.6%	(5.8%; 9.5%)
**Maternal HbA1c (at delivery)**						
<5.7 mmol/L	672	66.8%	(63.9%; 69.7%)	549	67.5%	(64.3%; 70.7%)
≥ 5.7 mmol/L	278	27.6%	(24.9%; 30.4%)	227	27.9%	(24.8%; 31.0%)
**Preeclampsia**						
No	977	97.1%	(96.1%; 98.2%)	794	97.7%	(96.6%; 98.7%)
Yes	29	2.9%	(1.8%; 3.9%)	19	2.3%	(1.3%; 3.4%)

^1^ Study population excludes multiple births (twins), subjects with missing inflammatory marker measurements or delivery mode information, and subjects with hs-CRP >200 μg/L

^2^ Restricted to vaginal spontaneous deliveries

**Table 2:** Characteristics of the UBCS study population.

	Total Population (N = 1090)			Study Population (n = 900) ^1^		
Factor	N	% or mean	(95%CI)	n	% or mean	(95%CI)
**Cord blood inflammatory markers**						
Adiponectin (mg/L)	1008	31.7	(30.9; 32.6)	900	32.1	(31.2; 33.0)
Leptin (μg/L)	1007	10.9	(10.1; 11.7)	900	10.7	(10.0; 11.4)
hs-CRP (μg/L)	922	52.4	(50.0; 54.8)	900	52.5	(50.1; 54.9)
**Delivery mode**						
Vaginal spontaneous	854	78.3%	(75.9%; 80.8%)	733	81.4%	(78.9%; 84.0%)
Elective cesarean	65	6.0%	(4.6%; 7.4%)	39	4.3%	(3.0%; 5.7%)
Emergency cesarean	126	11.6%	(9.7%; 13.5%)	95	10.6%	(8.5%; 12.6%)
Vaginal assisted	45	4.1%	(2.9%; 5.3%)	33	3.7%	(2.4%; 4.9%)
**Duration of labor (hours)**	1049	8.4	(8.0; 8.7)	883	8.4	(8.0; 8.8)
**Anesthesia (during delivery)** ^**2**^	207	24.2%	(21.4%; 27.1%)	169	23.1%	(20.0%; 26.1%)
**Induced labor delivery** ^**2**^	122	14.3%	(11.9%; 16.6%)	97	13.2%	(10.8%; 15.7%)
**Gender**						
Male	551	50.6%	(47.6%; 53.5%)	462	51.3%	(48.1%; 54.6%)
Female	537	49.3%	(46.3%; 52.2%)	438	48.7%	(45.4%; 51.9%)
**Gestational Age (weeks)**	1082	39.3	(39.2; 39.4)	894	39.4	(39.3; 39.5)
**Birth weight (g)**	1084	3379.1	(3350.9; 3407.3)	898	3413.3	(3383.3; 3443.2)
**Cord Blood pH**	1067	7.2	(7.2; 7.2)	884	7.2	(7.2; 7.2)
**Maternal age (years)**	1087	31.0	(30.7; 31.3)	899	30.9	(30.6; 31.3)
**Maternal nationality**						
Germany	867	79.5%	(77.1%; 81.9%)	712	79.1%	(76.5%; 81.8%)
Other	221	20.3%	(17.9%; 22.7%)	188	20.9%	(18.2%; 23.5%)
**Parity**						
0	541	49.6%	(46.7%; 52.6%)	453	50.3%	(47.1%; 53.6%)
1	399	36.6%	(33.7%; 39.5%)	329	36.6%	(33.4%; 39.7%)
> 1	141	12.9%	(10.9%; 14.9%)	111	12.3%	(10.2%; 14.5%)
**Maternal BMI (first trimester)**	1077	23.8	(23.5; 24.0)	891	23.8	(23.5; 24.1)
**Maternal education**						
> 11 years education	398	36.5%	(33.7%; 39.4%)	317	35.2%	(32.1%; 38.3%)
≤ 11 years education	662	60.7%	(57.8%; 63.6%)	559	62.1%	(58.9%; 65.3%)
**Maternal smoking during pregnancy**	161	14.8%	(12.7%; 16.9%)	131	14.6%	(12.3%; 16.9%)
**Preeclampsia**						
No	1073	98.4%	(97.7%; 99.2%)	888	98.7%	(97.9%; 99.4%)
Yes	17	1.6%	(0.8%; 2.3%)	12	1.3%	(0.6%; 2.1%)

^1^ Study population excludes multiple births (twins), subjects with missing inflammatory marker measurements or delivery mode information, and subjects with hs-CRP >200 μg/L

^2^ Restricted to vaginal spontaneous deliveries

**Fig 2:**
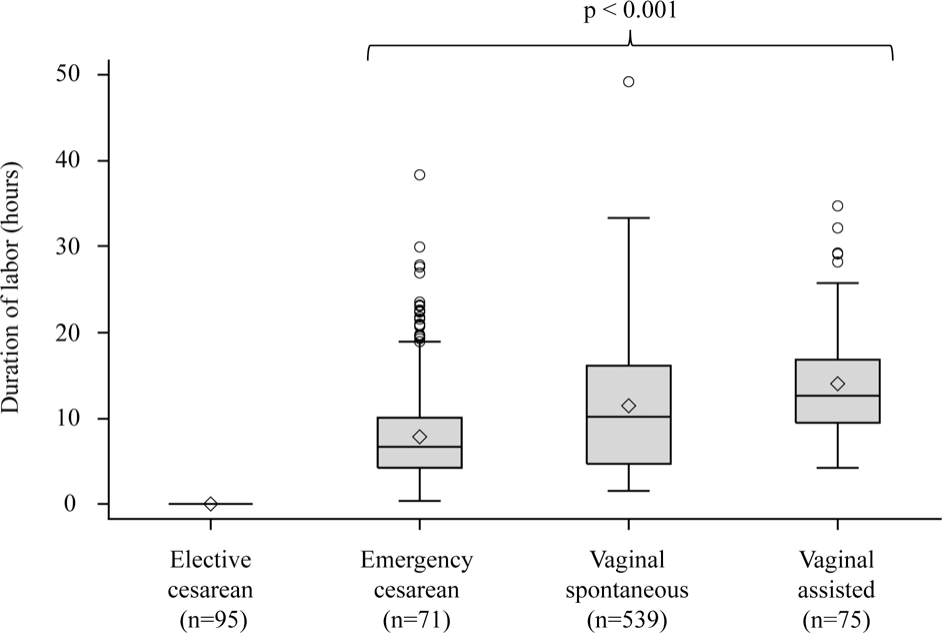
Duration of labor distribution by delivery mode in SPATZ. * Kruskal-Wallis p-value among active labor delivery modes; ◊ arithmetic mean value.

The interrelation of the two main explanatory variables, mode of delivery and duration of labor, is depicted in Figs 2 (SPATZ) and 3 (UBCS). Tables 3 (SPATZ) and 4 (UBCS) present associations of the main explanatory variables and potential confounders with each inflammatory marker. Lowest median concentrations of adiponectin, leptin, and hs-CRP were observed in elective cesarean delivery which was thus chosen as reference for subsequent models. Delivery mode and duration of labor were statistically significantly associated with leptin and hs-CRP and in SPATZ in addition with adiponectin. Gestational age and birthweight were confounders for all three inflammatory markers. Maternal lifestyle characteristics (first trimester BMI, education, and smoking status) were more closely associated with leptin and hs-CRP than with adiponectin. Among vaginal spontaneous deliveries, anesthesia during delivery and medically induced labor were associated with higher concentrations of leptin and hs-CRP while no significant association was found with adiponectin.

**Fig 3:**
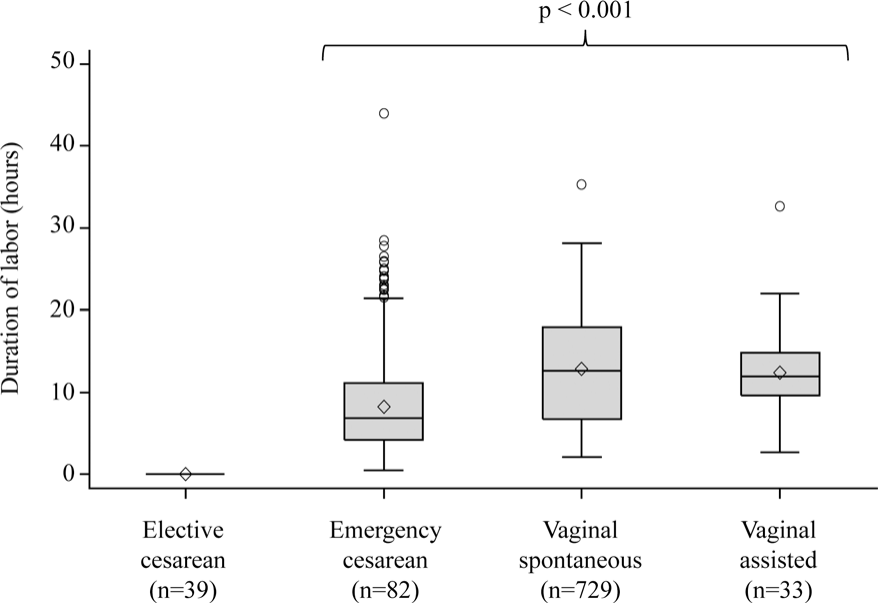
Duration of labor distribution by delivery mode in UBCS. * Kruskal-Wallis p-value among active labor delivery modes; ◊ arithmetic mean value.

**Table 3:** Bivariate associations with cord blood adiponectin, leptin, and hs-CRP in SPATZ.

	Median (p25, p75) or Spearman Coefficient and p-value ^1^						
Variable	n	Adiponectin		Leptin		hs-CRP	
**Delivery mode**			0.264		**0.003**		**< .001**
Vaginal spontaneous	547	30.3 (22.5, 39.6)		7.4 (4.4, 11.6)		30.6 (20.0, 47.4)	
Elective cesarean	95	27.1 (20.4, 38.3)		6.2 (3.6, 8.9)		23.9 (16.6, 38.3)	
Emergency cesarean	96	29.5 (19.8, 40.0)		8.1 (4.4, 20.6)		43.4 (23.1, 68.5)	
Vaginal assisted	75	28.3 (22.4, 37.8)		8.1 (5.3, 13.3)		35.5 (21.8, 55.2)	
**Duration of labor (hours)**	780	0.09	**0.013**	0.19	**< .001**	0.20	**< .001**
**Anesthesia (during delivery)** ^**2**^			0.060		0.205		**< .001**
No	413	31.2 (22.6, 40.4)		7.1 (4.1, 11.3)		28.3 (18.6, 44.0)	
Yes	134	29.1 (21.6, 36.1)		7.8 (4.9, 12.5)		36.5 (25.9, 59.2)	
**Induced labor delivery** ^**2**^			0.565		**0.010**		**0.015**
No	350	30.2 (22.6, 38.9)		6.9 (4.2, 10.9)		29.0 (18.6, 46.1)	
Yes	197	30.7 (22.4, 40.7)		8.8 (4.4, 12.8)		33.5 (21.9, 52.8)	
**Gender**			**0.005**		**< .001**		0.894
Male	432	28.7 (20.9, 37.8)		6.1 (3.4, 10.2)		29.9 (19.5, 50.6)	
Female	381	31.4 (22.9, 40.3)		8.8 (5.5, 13.9)		31.9 (21.0, 47.7)	
**Gestational Age (weeks)**	813	0.16	**< .001**	0.31	**< .001**	0.11	**0.002**
**Birth weight (g)**	813	0.18	**< .001**	0.48	**< .001**	0.05	0.147
**Cord Blood PH**	802	-0.00	0.946	-0.01	0.817	-0.15	**< .001**
**Base excess**	796	0.01	0.806	0.04	0.291	-0.04	0.306
**Maternal age (years)**	813	0.10	**0.004**	-0.00	0.971	-0.08	**0.022**
**Maternal nationality**			0.500		0.321		0.900
Germany	691	29.8 (22.1, 39.8)		7.1 (4.3, 11.7)		30.9 (19.9, 48.0)	
Other	113	30.2 (20.9, 37.0)		7.8 (4.7, 13.1)		30.1 (19.8, 52.0)	
**Parity**			0.773		0.570		**< .001**
0	426	29.8 (22.2, 39.2)		7.0 (4.5, 12.4)		33.5 (21.9, 53.0)	
1	291	30.1 (21.8, 39.7)		7.5 (4.2, 11.5)		26.9 (17.4, 45.8)	
> 1	96	29.1 (20.8, 38.5)		7.0 (3.7, 10.8)		31.6 (19.9, 45.6)	
**Maternal BMI (first trimester)**	789	-0.02	0.497	0.22	**< .001**	0.27	**< .001**
**Maternal education**			0.169		**0.034**		**< .001**
> 11 years education	477	30.9 (22.3, 39.5)		7.6 (4.7, 12.2)		27.9 (18.5, 44.5)	
≤ 11 years education	321	28.7 (21.5, 38.4)		6.9 (3.9, 11.4)		35.7 (22.6, 54.3)	
**Smoking during pregnancy**			0.232		**0.017**		0.128
No	734	30.2 (22.2, 39.0)		7.5 (4.5, 12.1)		30.4 (19.6, 48.6)	
Yes	62	26.0 (20.2, 40.7)		5.9 (3.2, 9.9)		35.5 (25.0, 50.9)	
**Maternal HbA1c (at delivery)**			0.493		**< .001**		**0.004**
<5.7 mmol/L	549	29.8 (21.3, 38.6)		6.9 (4.1, 11.2)		29.7 (19.1, 46.2)	
≥ 5.7 mmol/L	227	29.8 (22.6, 39.9)		8.8 (5.0, 14.5)		36.0 (22.2, 56.4)	
**Preeclampsia**			**0.043**		0.278		**0.013**
No	794	30.0 (22.0, 39.2)		7.2 (4.5, 11.9)		30.6 (19.7, 48.5)	
Yes	19	23.3 (13.6, 37.5)		6.5 (2.2, 12.8)		47.7 (29.3, 57.2)	

^1^ p-values reported as Kruskal-Wallis for categorical variables and Spearman Sum Rank for continuous variables

^2^ Restricted to vaginal spontaneous deliveries

**Table 4:** Bivariate associations with cord blood adiponectin, leptin, and hs-CRP in UBCS.

Factor	Median (p25, p75) or Spearman Coefficient and p-value ^1^						
	n	Adiponectin		Leptin		hs-CRP	
**Delivery mode**			0.144		**< .001**		**0.019**
Vaginal spontaneous	733	30.8 (22.5, 41.1)		7.9 (4.4, 13.3)		40.4 (25.8, 64.4)	
Elective cesarean	39	25.3 (20.3, 33.9)		4.5 (2.6, 7.9)		31.7 (24.6, 46.7)	
Emergency cesarean	95	29.3 (20.6, 38.3)		7.6 (4.2, 16.6)		46.3 (28.9, 82.7)	
Vaginal assisted	33	32.7 (24.0, 36.0)		6.7 (2.7, 10.8)		44.7 (30.8, 63.8)	
**Duration of labor (hours)**	883	0.03	0.369	0.14	**< .001**	0.19	**< .001**
**Anesthesia (during delivery)** ^**2**^			0.888		**0.037**		**< .001**
No	564	30.6 (22.5, 41.0)		7.8 (4.2, 12.5)		39.0 (25.4, 59.3)	
Yes	169	32.1 (22.4, 41.2)		8.6 (5.0, 15.5)		50.5 (30.0, 78.9)	
**Induced labor delivery** ^**2**^			0.053		0.181		**0.011**
No	636	31.2 (22.8, 41.3)		7.9 (4.3, 13.0)		39.7 (25.7, 61.5)	
Yes	97	27.0 (19.9, 39.6)		8.8 (5.0, 16.8)		47.8 (30.2, 77.2)	
**Gender**			0.237		**< .001**		0.997
Male	462	30.6 (21.4, 39.6)		6.1 (3.6, 10.7)		41.4 (26.5, 65.0)	
Female	438	30.5 (22.8, 41.2)		9.8 (5.3, 15.9)		40.1 (26.0, 65.6)	
**Gestation period (weeks)**	894	0.14	**< .001**	0.24	**< .001**	0.17	**< .001**
**Birth weight (g)**	898	0.12	**< .001**	0.40	**< .001**	0.08	**0.019**
**Cord Blood PH**	884	-0.05	0.148	-0.05	0.167	-0.11	**< .001**
**Base excess**	866	-0.03	0.328	0.01	0.775	-0.03	0.437
**Maternal age (years)**	899	-0.09	**0.007**	0.00	0.898	-0.04	0.229
**Maternal nationality**			0.143		0.283		0.101
Germany	712	30.4 (21.4, 40.2)		7.6 (4.2, 13.0)		39.9 (25.9, 64.4)	
Other	188	32.9 (24.3, 41.1)		8.1 (4.5, 14.0)		45.4 (28.8, 70.6)	
**Parity**			0.857		0.212		0.283
0	453	31.1 (21.5, 41.2)		8.0 (4.1, 13.4)		41.5 (26.8, 65.8)	
1	329	30.2 (22.0, 39.6)		7.3 (4.4, 12.3)		39.1 (25.8, 62.8)	
> 1	111	29.3 (23.5, 38.8)		8.4 (4.4, 15.6)		46.6 (25.8, 71.3)	
**Maternal BMI (first trimester)**	891	0.05	0.144	0.16	**< .001**	0.21	**< .001**
**Maternal education**			**0.028**		0.260		0.061
> 11 years education	317	29.1 (20.7, 39.3)		7.8 (5.0, 12.5)		38.9 (25.8, 59.8)	
≤ 11 years education	559	31.4 (23.0, 41.3)		7.5 (3.9, 13.3)		42.4 (27.0, 67.6)	
**Maternal smoking during pregnancy**			0.754		**0.017**		**0.035**
No	768	30.6 (22.3, 40.6)		7.9 (4.4, 13.3)		39.9 (26.0, 63.6)	
Yes	131	31.3 (22.3, 38.6)		6.2 (3.6, 12.3)		48.1 (27.2, 73.6)	
**Preeclampsia**			0.709		0.528		0.295
No	888	30.6 (22.3, 40.4)		7.8 (4.2, 13.2)		40.4 (26.0, 64.6)	
Yes	12	29.4 (20.6, 40.2)		7.1 (3.4, 10.9)		44.5 (31.6, 79.3)	

^1^ p-values reported as Kruskal-Wallis for categorical variables and Spearman Sum Rank for continuous variables

^2^ Restricted to vaginal spontaneous deliveries

Crude and adjusted model results are presented in Tables 5 (SPATZ) and 6 (UBCS). After adjustment, leptin and hs-CRP concentrations were higher among newborns delivered by active labor delivery vs. elective cesarean section. Associations were also statistically significant for all active delivery modes individually except for hs-CRP in UBCS which was confined to assisted vaginal deliveries. Increasing duration of labor was also statistically significantly associated with higher concentrations of leptin and hs-CRP. In models mutually adjusted for delivery mode and duration of labor, delivery mode was no longer associated with either leptin or hs-CRP whereas the association of duration of labor with these outcomes remained. No associations were observed between delivery mode or duration of labor and adiponectin concentrations in the adjusted models. Similar point estimates were observed for duration of labor in models restricted to active labor delivery modes using vaginal spontaneous labor as the reference group (data not shown).

**Table 5:** Crude and adjusted model results for adiponectin, leptin, and CRP by mode of delivery in SPATZ.

	Crude				Adjusted ^1^				Mutually Adjusted ^2^			
	Geometric Means Ratio (95% CI)		p-value	F-Test p-value ^3^	Geometric Means Ratio (95% CI)		p-value	F-Test p-value ^3^	Geometric Means Ratio (95% CI)		p-value	F-Test p-value ^3^
**Adiponectin**												
**Mode of Delivery**												
Elective Cesarean	1.00	reference			1.00	reference			1.00	reference		
Vaginal Spontaneous	1.06	(0.96; 1.17)	0.23	0.21	1.04	(0.93; 1.16)	0.50	0.40	0.98	(0.83; 1.15)	0.78	0.50
Emergency Cesarean	0.97	(0.86; 1.10)	0.67	0.21	0.96	(0.84; 1.10)	0.55	0.40	0.92	(0.75; 1.12)	0.38	0.50
Vaginal Assisted	1.01	(0.88; 1.15)	0.94	0.21	0.98	(0.84; 1.15)	0.84	0.40	0.91	(0.73; 1.13)	0.39	0.50
**Duration of Labor (per hour)**	1.04	(1.00; 1.07)	0.05	-	1.02	(0.98; 1.06)	0.41	-	1.03	(0.97; 1.10)	0.29	-
**Leptin**												
**Mode of Delivery**												
Elective Cesarean	1.00	reference			1.00	reference			1.00	reference		
Vaginal Spontaneous	1.15	(0.95; 1.40)	0.15	0.02	**1.27**	**(1.07; 1.50)**	**0.006**	0.002	1.05	(0.82; 1.35)	0.69	0.40
Emergency Cesarean	**1.46**	**(1.13; 1.88)**	**0.003**	0.02	**1.44**	**(1.16; 1.73)**	**<0.001**	0.002	1.19	(0.88; 1.61)	0.25	0.40
Vaginal Assisted	1.28	(0.98; 1.67)	0.07	0.02	**1.49**	**(1.17; 1.89)**	**0.001**	0.002	1.17	(0.84; 1.63)	0.36	0.40
**Duration of Labor (per hour)**	**1.15**	**(1.07; 1.23)**	**<0.001**	-	**1.14**	**(1.07; 1.21)**	**<0.001**	-	**1.10**	**(1.00; 1.21)**	**0.05**	-
**CRP**												
**Mode of Delivery**												
Elective Cesarean	1.00	reference			1.00	reference			1.00	reference		
Vaginal Spontaneous	**1.20**	**(1.03; 1.40)**	**0.02**	< .001	**1.28**	**(1.07; 1.52)**	**0.006**	< .001	0.97	(0.75; 1.26)	0.83	0.44
Emergency Cesarean	**1.61**	**(1.32; 1.98)**	**<0.001**	< .001	**1.58**	**(1.29; 1.94)**	**<0.001**	< .001	1.12	(0.82; 1.53)	0.47	0.44
Vaginal Assisted	**1.42**	**(1.14; 1.76)**	**0.002**	< .001	**1.47**	**(1.15; 1.89)**	**0.002**	< .001	1.04	(0.74; 1.47)	0.82	0.44
**Duration of Labor (per hour)**	**1.16**	**(1.10; 1.22)**	**<0.001**	-	**1.16**	**(1.09; 1.24)**	**<0.001**	-	**1.15**	**(1.04; 1.27)**	**0.005**	-

^1^ Models adjusted for gender, gestational age, birthweight, pre-pregnancy BMI, education, smoking, cord blood pH, base excess, and HbA1c

^2^ Mutually adjusted models include all above covariates and log transformed duration of labor.

^3^ P-value for combined delivery modes (i.e. active labor) against the reference category calculated as probability of the F-statistic

**Table 6:** Crude and adjusted model results for adiponectin, leptin, and CRP by mode of delivery in UBCS.

	Crude				Adjusted ^1^				Mutually Adjusted ^2^			
	Geometric Means Ratio (95% CI)		p-value	F-Test p-value ^3^	Geometric Means Ratio (95% CI)		p-value	F-Test p-value ^3^	Geometric Means Ratio (95% CI)		p-value	F-Test p-value ^3^
**Adiponectin**												
**Mode of Delivery**												
Elective Cesarean	1.00	reference			1.00	reference			1.00	reference		
Vaginal Spontaneous	**1.18**	**(1.01; 1.38)**	**0.040**	0.144	1.07	(0.90; 1.27)	0.419	0.555	1.06	(0.86; 1.30)	0.611	0.549
Emergency Cesarean	1.11	(0.93; 1.34)	0.247	0.144	1.00	(0.82; 1.21)	0.996	0.555	0.97	(0.76; 1.25)	0.840	0.549
Vaginal Assisted	1.10	(0.88; 1.38)	0.391	0.144	1.06	(0.83; 1.35)	0.634	0.555	1.04	(0.78; 1.38)	0.795	0.549
**Duration of Labor (per hour)**	1.03	(0.99; 1.08)	0.164	-	1.01	(0.96; 1.06)	0.763	-	1.01	(0.95; 1.07)	0.824	-
**Leptin**												
**Mode of Delivery**												
Elective Cesarean	1.00	reference			1.00	reference			1.00	reference		
Vaginal Spontaneous	**1.74**	**(1.32; 2.31)**	**< .001**	< .001	**1.56**	**(1.20; 2.01)**	**< .001**	0.001	1.23	(0.90; 1.68)	0.198	0.068
Emergency Cesarean	**1.95**	**(1.41; 2.69)**	**< .001**	< .001	**1.75**	**(1.31; 2.34)**	**< .001**	0.001	1.39	(0.96; 2.01)	0.082	0.068
Vaginal Assisted	1.41	(0.94; 2.11)	0.096	< .001	1.29	(0.89; 1.86)	0.176	0.001	0.96	(0.63; 1.47)	0.852	0.068
**Duration of Labor (per hour)**	**1.21**	**(1.12; 1.31)**	**< .001**	-	**1.18**	**(1.10; 1.27)**	**< .001**	-	**1.14**	**(1.04; 1.25)**	**0.006**	-
**CRP**												
**Mode of Delivery**												
Elective Cesarean	1.00	reference			1.00	reference			1.00	reference		
Vaginal Spontaneous	1.18	(0.96; 1.45)	0.111	0.021	1.13	(0.91; 1.41)	0.252	0.087	0.89	(0.68; 1.15)	0.372	0.391
Emergency Cesarean	**1.37**	**(1.08; 1.73)**	**0.010**	0.021	1.23	(0.96; 1.56)	0.100	0.087	0.90	(0.66; 1.23)	0.525	0.391
Vaginal Assisted	**1.41**	**(1.05; 1.89)**	**0.023**	0.021	**1.42**	**(1.05; 1.93)**	**0.024**	0.087	1.05	(0.73; 1.50)	0.792	0.391
**Duration of Labor (per hour)**	**1.16**	**(1.10; 1.23)**	**< .001**	-	**1.12**	**(1.06; 1.19)**	**< .001**	-	**1.14**	**(1.05; 1.23)**	**0.001**	-

^1^ Models adjusted for gender, gestational age, birthweight, pre-pregnancy BMI, education, smoking, cord blood pH, base excess, and HbA1c

^2^ Mutually adjusted models include all above covariates and log transformed duration of labor.

^3^ P-value for combined delivery modes (i.e. active labor) against the reference category calculated as probability of the F-statistic

Following restriction to vaginal spontaneous deliveries, the associations of duration of labor with leptin and hs-CRP remained similar [SPATZ: GMR (95%CI): 1.10 (0.99; 1.22) and 1.10 (0.99; 1.23); UBCS: 1.12 (1.02; 1.23) and 1.12 (1.03; 1.22); per hour of labor respectively]. In SPATZ, medically induced labor was associated with higher concentrations of hs-CRP [1.17 (1.01; 1.36)]. These point estimates were slightly stronger following mutual adjustment. Administration of anesthesia had no effect on any inflammatory marker. This was reversed in UBCS where only administration of anesthesia was marginally associated with higher concentrations of hs-CRP [1.12 (1.00; 1.25)]. However, in mutually adjusted models this effect was attenuated.

## Discussion

Though several previous studies have assessed cesarean delivery as a potential confounder in the association between inflammatory markers and disease[28], we found only three studies that primarily investigated association between both pre- and post-onset of labor delivery modes and either adiponectin, leptin, or CRP measured in either cord blood or newborn serum. Our results were mostly consistent with associations reported for lower leptin[24] and CRP concentrations[23] among elective cesarean deliveries. Although, our results did not support the findings of Hermansson et al. who reported significant association between elective cesarean birth and lower concentrations of adiponectin.[22]

Mean adiponectin as well as leptin concentrations were higher in our study populations than those reported in other studies for elective cesarean and vaginal delivery.[22,24] However, between our two cohorts the distributions of the biomarker concentrations were very similar. The aforementioned differences were likely attributable to differences in study populations and unlikely to have influenced results from adjusted models after accounting for a number of demographic confounders.

The results of our study may have been influenced by the following factors. First, we observed a consistent decrease in adiponectin concentration results with increasing sample storage duration in SPATZ likely attributable to long-term storage effect. To account for this, we standardized on storage duration. Second, duration of labor was imputed as zero for elective cesarean delivery and calculated using self-reported onset of labor for active deliveries. Mutually adjusted models restricted to active labor deliveries showed no change in point estimate for duration of labor indicating imputed zero duration did not influence full model results (data not shown). Similarly, results from sensitivity analyses excluding subjects with extreme or suspicious labor durations were not significantly different from among all subjects (data not shown). Third, we could not fully assess whether cord blood inflammatory markers were influenced by subclinical infections for which treatment was not prescribed. In order to reduce this possibility, our study population excluded newborns transferred to intensive care immediately after delivery and the analysis was further restricted to children with hs-CRP levels below 200 μg/L.[27] Finally, our study populations included few women who had preeclampsia which has been associated with higher cord blood leptin concentrations.[29] Exclusion of these women did not alter the observed associations (data not shown).

In contrast to previous efforts, our analysis has notable strengths. Our study populations were much larger, allowing control for multiple confounding variables. Furthermore, we were able to analyze the effects of duration of labor, anesthesia, and induction as potential mediators of the association with delivery mode. Moreover, our analyses were aided by our ability to classify vaginal deliveries into “spontaneous” and “assisted” subgroups between which duration of labor was clearly different. Finally, we were able to demonstrate very similar findings in two independent cohorts conducted with similar if not identical methodology. Of note, shifts in the prevalences of several demographic, lifestyle, and delivery-related factors occurred between both studies. In SPATZ, mothers more often had elective cesarean delivery, medical induction of labor, and administration of anesthesia. Furthermore, maternal smoking prevalence during pregnancy decreased, maternal age and education increased, and maternal BMI before pregnancy slightly increased over time. Also, in the later Ulm SPATZ Health Study we observed similar overall adiponectin and leptin concentrations and a similar but shifted hs-CRP distribution. Replication despite these differences strengthens our findings.

Our results imply that duration of labor rather than delivery mode may be primarily responsible for increased concentrations of leptin and hs-CRP. Our results support those of a smaller Korean study which reported correlation between duration of labor and cord blood leptin concentration among vaginal spontaneous deliveries.[30] Though it remains unclear why leptin levels may increase during labor, previous research suggests adiponectin and leptin secreted by both the placenta and fetal adipocytes may signal release of pro-inflammatory cytokines which are then thought to stimulate production of CRP in placental tissue during labor.[31,32]

Although we were able to control for some prenatal factors by including gestational age and birthweight in our models, we could not fully account for all factors that may be associated with duration of labor and neonatal inflammation. Therefore, it remains unclear if duration of labor is part of the causal pathway for previously reported associations between cord blood inflammatory markers and disease or if it is simply a proxy for other unmeasured factors present during or prior to delivery. Subsequent analyses should consider the effect of additional factors, such as fetal growth and placental defects on levels of inflammatory biomarkers in cord blood. Also, administration of anesthesia and medical induction of labor may warrant further investigation given the mixed findings in our studies and the limited ability to explore these factors among assisted vaginal and emergency cesarean sections due to our sample sizes. Moreover, further cytokines should be investigated to understand the kinetics of the inflammatory response and the inflammatory profile potentially triggered by duration of labor.

Our results may benefit clinical practice where CRP screening is a common tool for diagnosing neonatal infection. Current guidelines for interpreting CRP concentrations are vague, leading to wide variation in treatment strategies among physicians.[33] Given our findings, future studies seeking standardized methods of interpreting CRP as a biomarker for infection should include duration and induction of labor as potential confounding variables.

## Conclusion

In this study, we observed that cord blood leptin, and hs-CRP concentrations were primarily associated with duration of labor rather than delivery mode. Further research is warranted to investigate surrounding factors and whether duration of labor may explain associations observed between mode of delivery and health outcomes later in life. Evaluation of these factors may also improve the interpretation of CRP as a biomarker for infection in the neonate.
